# The Role of Molecular Flexibility in Antigen Presentation and T Cell Receptor-Mediated Signaling

**DOI:** 10.3389/fimmu.2018.01657

**Published:** 2018-07-17

**Authors:** Kannan Natarajan, Jiansheng Jiang, Nathan A. May, Michael G. Mage, Lisa F. Boyd, Andrew C. McShan, Nikolaos G. Sgourakis, Ad Bax, David H. Margulies

**Affiliations:** ^1^Molecular Biology Section, Laboratory of Immune System Biology, National Institute of Allergy and Infectious Diseases, National Institutes of Health, Bethesda, MD, United States; ^2^Department of Chemistry and Biochemistry, University of California at Santa Cruz, Santa Cruz, CA, United States; ^3^Laboratory of Chemical Physics, National Institute of Diabetes and Digestive and Kidney Diseases, National Institutes of Health, Bethesda, MD, United States

**Keywords:** major histocompatibility complex, T cell receptor, tapasin, transporter associated with antigen presentation, TAP-binding protein, related, chaperone

## Abstract

Antigen presentation is a cellular process that involves a number of steps, beginning with the production of peptides by proteolysis or aberrant synthesis and the delivery of peptides to cellular compartments where they are loaded on MHC class I (MHC-I) or MHC class II (MHC-II) molecules. The selective loading and editing of high-affinity immunodominant antigens is orchestrated by molecular chaperones: tapasin/TAP-binding protein, related for MHC-I and HLA-DM for MHC-II. Once peptide/MHC (pMHC) complexes are assembled, following various steps of quality control, they are delivered to the cell surface, where they are available for identification by αβ receptors on CD8^+^ or CD4^+^ T lymphocytes. In addition, recognition of cell surface peptide/MHC-I complexes by natural killer cell receptors plays a regulatory role in some aspects of the innate immune response. Many of the components of the pathways of antigen processing and presentation and of T cell receptor (TCR)-mediated signaling have been studied extensively by biochemical, genetic, immunological, and structural approaches over the past several decades. Until recently, however, dynamic aspects of the interactions of peptide with MHC, MHC with molecular chaperones, or of pMHC with TCR have been difficult to address experimentally, although computational approaches such as molecular dynamics (MD) simulations have been illuminating. Studies exploiting X-ray crystallography, cryo-electron microscopy, and multidimensional nuclear magnetic resonance (NMR) spectroscopy are beginning to reveal the importance of molecular flexibility as it pertains to peptide loading onto MHC molecules, the interactions between pMHC and TCR, and subsequent TCR-mediated signals. In addition, recent structural and dynamic insights into how molecular chaperones define peptide selection and fine-tune the MHC displayed antigen repertoire are discussed. Here, we offer a review of current knowledge that highlights experimental data obtained by X-ray crystallography and multidimensional NMR methodologies. Collectively, these findings strongly support a multifaceted role for protein plasticity and conformational dynamics throughout the antigen processing and presentation pathway in dictating antigen selection and recognition.

## Dedication

In recognition of William E. Paul’s personal encouragement to explore new approaches to address fundamental aspects of the immune response, we offer this review that reflects recent progress in studies of antigen presentation and T cell receptor-mediated signaling. Dr. Paul’s commitment to rigorous analysis and quantitative experimentation continues to serve as a paradigm for our research.

## Introduction

Experimental approaches to solving fundamental problems in immunology range from the biological to the biophysical, exemplified by early observations concerning immunity to infection and chemical and biochemical studies of toxins, blood groups, haptens, and antibodies. Contemporary molecular biological and structural studies of antibodies, major histocompatibility complex (MHC) molecules, Fc receptors, and T cell receptors (TCRs), as well as many other immunologically relevant molecules, not only expand our understanding of the immune system but also have been instrumental in developing methodologies with broader application (1). Central to the immune response are the cellular pathways of antigen processing and presentation—the mechanisms by which peptides derived from foreign or self proteins are degraded into peptides of appropriate length and are then captured by MHC class I (MHC-I) or MHC class II (MHC-II) molecules which display these peptide fragments as peptide/MHC (pMHC) complexes at the surface of antigen-presenting cells (APC) (2, 3). Such pMHC complexes are then available for identification by T cells, which are subsequently activated to initiate various cellular programs. These may result in cytolysis of target cells (primarily by CD8^+^, MHC-I-restricted T cells) or production of various cytokines (by either CD8^+^ or by CD4^+^, MHC-II-restricted T cells) that direct, coordinate, and induce further immunological responses such as antibody production by B cells or differentiation into memory T cells. Cell surface MHC-I molecules may also interact with various inhibitory, and in some cases activating, natural killer (NK) cell receptors, and thus contribute to a regulatory role in the NK arm of the innate immune response (4–6). Various genetic, molecular biological and structural approaches have examined peptide–protein and protein–protein interactions that are necessary to generate an immune response. Our primary goal in this review is to highlight the role of molecular flexibility in governing molecular interactions required for antigen processing, presentation, and recognition, as illustrated by the function of MHC molecules, their chaperones, and TCR in antigen presentation and recognition. Recent reviews have summarized aspects of this flexibility, largely based on computational approaches (7, 8). Our emphasis here will be on recent experimental observations based on X-ray crystallography and nuclear magnetic resonance (NMR) spectroscopy (9–15).

Much of our current understanding of protein structure has been revealed by X-ray crystallography, a technique that is unrivaled in its ability to provide high resolution structural details (16–19). X-ray data often reveal regions of proteins that are found in poor electron density, or that exhibit high values of the crystallographic *B-* factor, indications of flexible or dynamic parts of the molecule (20). Computational molecular dynamics (MDs) and normal mode analysis, based on X-ray structures, provide predictive approaches to visualizing protein flexibility (7, 21, 22). However, the most informative experimental elucidation of dynamic regions of proteins comes from NMR spectroscopy. NMR analysis of proteins in solution provides information on conformational changes over time scales ranging from picoseconds to days, thus encompassing dynamics ranging from bond vibrations to side chain flips to large scale domain motions, and the residue-specific stability of H-bonds (23). NMR is also powerful because it can characterize sparsely populated (i.e., transient) conformational states that may be important for biological function (24). Contemporary protein-labeling and multidimensional NMR techniques permit examination of protein complexes as large as 1 MDa (25, 26). In addition, all atom MD simulations may complement the experimental NMR and contribute to elucidating such dynamic processes. The discussion below focuses on the dynamics of proteins involved in antigen presentation, largely based on experimental analyses.

In this review, we will explore the dynamics of pMHC with respect to three aspects of antigen presentation: (1) the formation of the tri-molecular complex consisting of peptide, and MHC [for MHC-I, peptide, MHC-I heavy chain, and the light chain, β2-microgolobulin (β_2_m)] as inferred from numerous X-ray structures and recent NMR analyses; (2) the influence of the pMHC chaperones, tapasin and TAP-binding protein, related (TAPBPR) for MHC-I and HLA-DM (H2-DM in the mouse) for MHC-II; and (3) alterations of the conformational dynamics of the TCR upon pMHC interaction that reflect early steps in TCR-mediated signaling. Our focus is on MHC-I, but we will describe analogous steps in the MHC-II processing and presentation pathway as well. Our discussion of peptide, MHC-I, MHC-II, and TCR dynamics follows brief summaries of the major steps of MHC antigen processing and presentation.

## Major Steps in MHC Antigen Processing and Presentation: MHC-I

**Figure 1:**
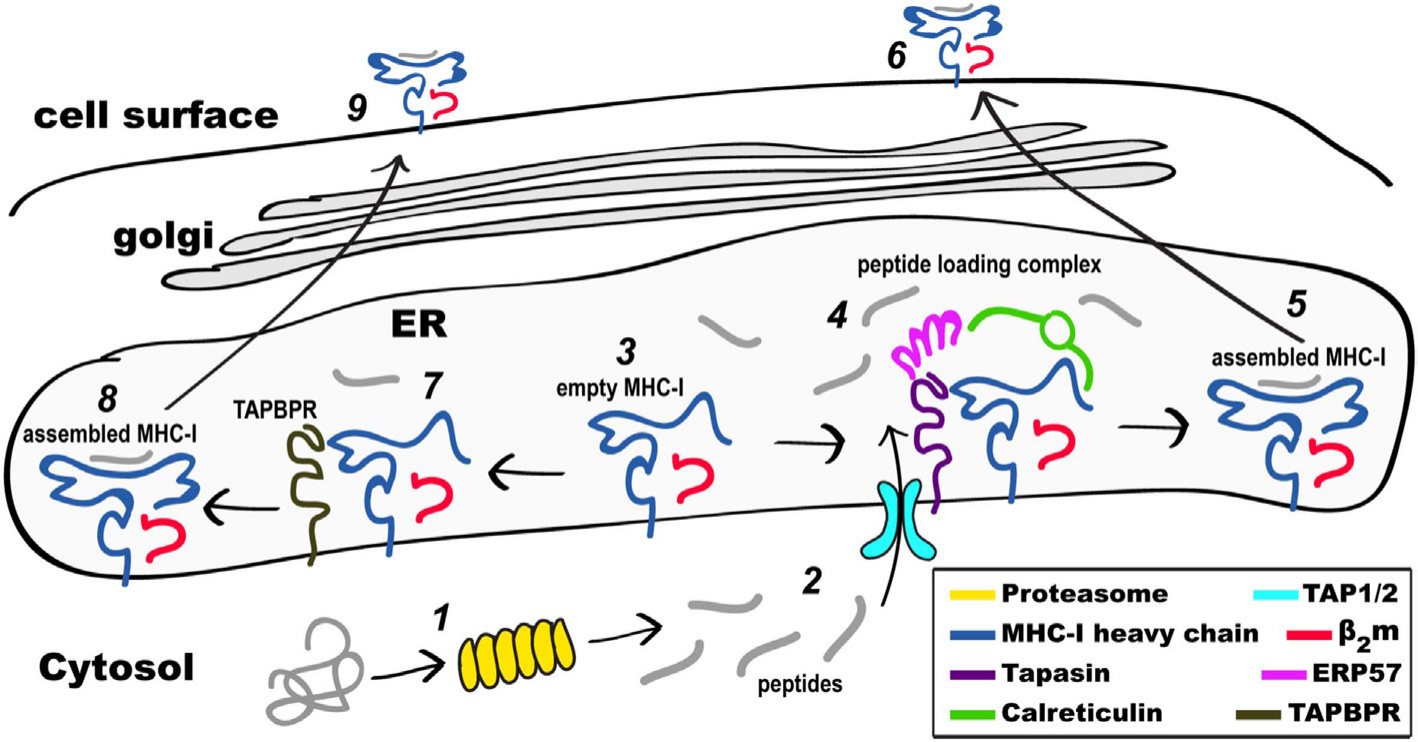
Schematic view of MHC class I (MHC-I) pathways of antigen processing and presentation. Proteins in the cytosol engage the proteasome (1) and the peptides generated (2) are transported through the TAP1/2 transporter to the endoplasmic reticulum (ER). Partially folded MHC-I/β2-microgolobulin (β_2_m) complexes (3) are stabilized as part of the peptide loading complex (PLC) (4) where they may be retained in a peptide-receptive state. Once high affinity peptide is bound, the peptide/MHC-I (5) is released from the PLC and destined for the secretory pathway and the cell surface (6). Alternatively, partially folded MHC-I/β_2_m complexes (3) are stabilized by interaction with TAP-binding protein, related (TAPBPR) (7), loaded with peptide, released from TAPBPR (8), and the assembled MHC-I proceeds to the cell surface (9). Not illustrated are the peptide trimming enzymes (ERAAP or ERAP1/2) or the quality control UGGT1 interaction as described in the text.

The cellular and molecular bases by which peptides are generated by the proteasome in the cytoplasm, transported *via* transporter associated with antigen presentation (TAP) to the endoplasmic reticulum (ER), where they are loaded onto nascent MHC-I, have been the focus of considerable attention for several decades, and a number of reviews address this process (2, 8, 27–31). Here, we summarize the process and the critical steps, with a focus on MHC-I, as shown schematically in Figure 1. MHC-II follows a similar but distinct process (32, 33).

The antigenic peptides bound by MHC-I in general derive from proteins located in the cytoplasm, proteins that are degraded by the proteasome following unfolding or misfolding and ubiquitination, or proteins that result from aberrancies in translation initiation, mRNA splicing, or alternate reading frames (34, 35). The sources of these peptides may be self-proteins, sometimes expressed at abnormal levels in cancer cells, or pathogen-derived products expressed following infection. These peptide products of the proteasome must then be transported to the site of MHC-I folding, assembly and maturation, the ER, a function provided by the heterodimeric transporter associated with antigen processing, TAP (29, 36). In the ER, peptides load onto MHC-I following motif rules during the folding process, are trimmed by aminopeptidases, and their binding is monitored by quality control mechanisms in the ER and Golgi. For MHC-I, limits on the preferred length of antigenic peptides are imposed by a binding cleft with closed ends, and peptides, usually of 8–12 amino acids in length, are generated by the progressive results of proteasome degradation, length limits for TAP transport, and amino-terminal trimming by endoplasmic reticulum aminopeptidase 1 (ERAP1).

MHC class I heavy chains, the “human leukocyte antigens,” denoted HLA-A, -B, or -C in the human, H2 in the mouse, are ~40 kDa glycoproteins that exhibit the greatest genetic polymorphism known. Presently, some 13,000 HLA class I and almost 5,000 HLA class II alleles are recognized (https://www.ebi.ac.uk/ipd/imgt/hla/stats.html). The MHC-I heavy chain, a type I membrane glycoprotein, assembles with the monomorphic light chain, β_2_m, and a peptide, usually of 8–12 amino acids in length, taken from the cell’s ER peptide pool (Figure 1). Peptides are bound *via* anchor positions that engage pockets of the MHC, designated A–F (37). Any “single” MHC molecule, purified from a cellular source, can associate with an ensemble of hundreds or thousands of self-peptides (38). The first crystal structure of an HLA-A2 molecule was based on a heterogeneous peptide/HLA-2/β_2_m preparation and thus electron density corresponding to the peptide was poorly defined (39). Numerous subsequent X-ray structures have been determined based on methods for producing homogeneous complexes employing various expression and purification strategies (40).

Intracellularly, the MHC-I protein is synthesized on membrane-bound ribosomes and delivered vectorially into the lumen of the ER, where initial folding, including formation of the intrachain disulfide bond of the membrane proximal α3 domain, along with assembly to the β_2_m light chain, takes place. A molecular chaperone, calnexin (41, 42), stabilizes the partially folded regions of MHC-I until the heavy chain engages the peptide loading complex (PLC), which consists of the heterodimeric TAP1/2 peptide transporter (43), tapasin (44), a chaperone that stabilizes peptide receptive (PR) MHC-I, ERp57, a tapasin-associated oxidoreductase (45), and calreticulin (46). Peptides load onto PR MHC-I in the PLC, and trimming of their amino termini is accomplished by ER-associated amino peptidase [known as ERAAP in the mouse (47) or ERAP1/2 in the human (48)]. Recently, cryo-electron microscopic images of the full PLC purified from a human lymphoblastoid cell line have been obtained (49), revealing a multimolecular complex containing the two-pseudo-symmetric editing modules, centered around the TAP transporter, consistent with previous biochemically derived structural models (27, 50). Once high affinity peptide is loaded onto MHC-I, the pMHC/β_2_m complex is released and then proceeds through the Golgi where quality control based on carbohydrate composition occurs (51, 52). The acquisition of high-affinity peptide by MHC-I is assured by the coordinated functions of the proteins of the PLC, in particular tapasin. In recent years, a tapasin homolog, TAPBPR, has been recognized as a molecule with similar function to the PLC, but that accomplishes its role independent of the PLC and its associated components (53). Assembled, stable, peptide/MHC-I (pMHC-I) complexes are then displayed at the cell surface.

## Major Steps in MHC Antigen Processing and Presentation: MHC-II

The folding, assembly and peptide-loading of MHC-II molecules, though similar in some respects to that of MHC-I, occurs in distinct cellular compartments and is focused on binding peptides generated not from an “inside-out” pathway like MHC-I, but rather from those produced from proteins that derive from the extracellular environment (2, 33). Thus, proteins taken up by endocytosis or phagocytosis enter the endocytic pathway where they are proteolyzed and denatured, and where they encounter MHC-II molecules, consisting of previously assembled complexes consisting of α and β chains bound to the Ii (invariant chain). Processing of Ii, release of the CLIP peptide derived from it, and concomitant interaction with HLA-DM, a peptide-exchange catalyst/chaperone, result in peptide-loaded (PL) MHC-II that then go to the cell surface for recognition by CD4^+^ T cells (54–56). The role of HLA-DM in optimizing the class II peptide repertoire parallels the role of the PLC or TAPBPR in the MHC-I peptide loading pathway (57).

## Peptide Dynamics

Dynamics of peptides bound to MHC molecules have been the focus of both experimental and computational studies that have been recently reviewed (58). Characterizing peptide conformational plasticity and dynamics within the MHC groove is of considerable interest because peptides: (1) influence MHC thermal and kinetic stability as well as the structural ensembles and free energy landscape of the assembled MHCs and (2) play a key role in recognition by TCR and NK receptors (NKR). These features of molecular flexibility of peptides are important for a proper immune response and impact MHC cell surface lifetime, receptor recognition and antigen immunogenicity. Exactly how peptide dynamics regulate antigen processing and presentation is an ongoing field of study.

Association of the TCR with pMHC molecules often induces localized conformational changes in the backbone and side chain of the bound peptide (59). It is hypothesized that if the peptide is presented by the MHC with a conformation and surface chemistry that is not optimized for TCR recognition, the pMHC will exhibit slow TCR binding, relative to a peptide presented in a more restricted, pre-optimized conformation. During this antigen recognition process, peptide motions impact the formation of complementarity pMHC/TCR interaction interfaces, in terms of both shape and chemical composition. The timescale of the peptide motions contributes to the affinity of pMHC/TCR recognition by imposing energetic and kinetic barriers for complex formation, and stability of the resulting complex. Initial insights into this phenomenon were obtained from a comparison of the X-ray conformations of the HTLV-1 derived Tax_11–19_ peptide bound to HLA-A2 in the presence or absence of a high affinity TCR indicated an induced fit of the peptide of the pMHC complex when bound to the TCR (58). In these structures, the conformational change in Tax_11–19_ upon TCR binding is highlighted by significant rearrangements of the backbone atoms of Pro6 and Val7. NMR analyses of the ^15^N- and ^13^C-labeled Tax_11–19_ peptide bound to HLA-A2 revealed multiple resonances for Val7 of the peptide reflecting a slower than millisecond timescale of interconversion between alternate peptide conformations. In this example, the crystallographic suggestion of conformational plasticity of an MHC-I-bound peptide has been reinforced by the behavior in solution as detected by NMR.

Multiple peptide conformations have also been observed in the well characterized QL9/H2-L^d^ model system, where NMR analyses revealed two conformations of the bound 9-mer QL9 peptide as indicated by the presence of two unique chemical shifts in slow-exchange for the amide resonance of Phe7 of the peptide in the MHC-bound state (60). Intriguingly, Phe7 was reported to remain conformationally mobile even when interfacing with the CDR3β loop of the cognate 2C TCR. Matching conformational dynamics between receptor and ligand has been proposed as a mechanism to enhance the thermodynamic stability of pMHC/TCR complexes (60). This may result from reducing the entropic penalty associated with restraining otherwise flexible surfaces and reflects the enhanced stability of what might otherwise be a weak TCR/pMHC complex.

An illustration of the dynamic nature of pMHC-II molecules was seen in the pigeon cytochrome *c* (PCC) 91–104 peptide/I-E^k^ (pMHC-II) model system in which two distinct conformations of a bound ^19^F-labeled peptide were observed by NMR (61). The two peptide conformations corresponded to kinetically distinct species of PCC91–104/I-E^k^ complexes identified by their fast and slow dissociation rates (62). Careful studies of MHC-II molecules binding peptides displayed in alternate registers reveal potential complexities that may result from the peptide binding groove being open at both ends (63). Conformational isomers of the same peptide presented by the same MHC-II molecules have been identified based on distinct T cell reactivities (64). Indeed, one study employing spin-labeled peptide and NMR analysis demonstrated that an MHC-II-restricted peptide can bind in either the canonical N to C (left to right) or flipped (right to left) conformation (65).

## “Empty” MHC-I Molecules

Peptides bound in the groove of MHC-I and MHC-II molecules serve two indispensable and interrelated functions: (1) to form part of a composite pMHC ligand recognized by T and NK cell receptors and (2) to structurally stabilize MHC molecules for long-lived display at the cell surface. However, under certain physiological conditions “empty” or peptide-free conformers of MHC-I occur at the cell surface as detected by specific monoclonal antibodies or by peptide binding assays. The LA45 monoclonal antibody reacts with a β_2_m-free form of human HLA molecules on phytohemagglutinin-activated human mononuclear leukocytes and on transformed cell lines (66). Similarly, in the mouse, the 64-3-7 antibody recognizes peptide free forms of H2-L^d^ in cellular lysates and at the cell surface (67). Peptide-binding experiments indicate the presence of empty, peptide-receptive HLA-B27 molecules on the cell surface (68), perhaps contributing to the etiology of HLA-B27-associated arthritic disease. A functional role for empty MHC-I molecules at the cell surface in modulating immune responses was inferred from early studies (69–72). A recent report identified empty HLA-B*35:01 molecules on activated T cells and showed preferential binding of such alternatively conformed structures to CD8 resulting in enhanced T cell responses (73).

MHC class I molecules devoid of, or bearing low affinity, peptides fail to reach the cell surface efficiently at physiological temperature, but can be detected if the cells are incubated at room temperature (74). The distinct conformation of such molecules may be discerned by comparing the reactivity of monoclonal antibodies that detect peptide-independent and peptide-dependent epitopes (67, 75, 76). These “empty” MHC-I molecules result from genetic lesions in the peptide-loading steps of the antigen presentation pathway, specifically in major components of the PLC including TAP and tapasin (77, 78).

Some non-classical MHC-I-like molecules, such as human HLA-F can be expressed as either peptide-free (open-conformer) or PL forms. Such molecules may differentially interact with either activating or inhibitory NKR to innate immune responses (79–81). Understanding the structural contributions of peptide to fully loaded MHC-I and MHC-II molecules provides insight into the mechanisms involved in peptide loading and exchange. However, the instability of peptide-free molecules has precluded crystallographic studies of these molecules.

Nuclear magnetic resonance methods are especially well-suited to analyzing conformational dynamics in MHC-I molecules since these proteins are routinely prepared by bacterial expression thus permitting uniform labeling with the desired isotope (82). In addition, the heavy and light chains can be separately labeled, greatly improving spectral resolution. NMR analysis of MHC-II molecules has been hampered by the difficulty in producing these proteins by bacterial expression, although several groups have reported success in this area (55, 83).

The MD and structural features of peptide-free MHC molecules are of key importance for understanding the mechanism of peptide loading as peptide-free forms of MHC molecules are substrates for peptide loading and exchange by chaperones such as tapasin and TAPBPR for MHC-I and HLA-DM for MHC-II. However, it is challenging to produce peptide-free MHC-I molecules in amounts sufficient for detailed structural analyses and therefore information regarding their conformational dynamics has been largely obtained from MD stimulations [see, for example, Ref. (84, 85)]. An early biophysical and structural analysis of a peptide-free HLA-B*0702/β_2_m heterodimer described an unstable, partially unfolded molecule in a molten globule state (86). More recently Kurimoto et al. (87) applied solution NMR techniques to peptide-free HLA-C*07:02/β_2_m. NMR spectra obtained by selective labeling of methionine residues in the heavy chain revealed markedly attenuated intensities for residues in the peptide-binding domain suggestive of a partially folded molten globule form, whereas the α3 domain was properly conformed. These experiments highlight the role of the bound peptide in stabilizing MHC conformations for display at the cell surface to function as ligands for T cell and NK cell receptors.

## Dynamics of pMHC-I

Although crystal structures of MHC-I molecules encompassing various allelomorphs and peptides show little gross variation, their analyses in solution by various biophysical methods and MD simulations indicate considerable differences in molecular flexibility at localized regions (7). Recent NMR analyses of pMHC-I complexes show heavy chain backbone as well as methyl side-chain dynamics revealing flexibility in exposed loops of the platform domain of the molecule (11).

The contribution of MHC-conformational dynamics to the relative dependence of MHC-I molecules on tapasin chaperone function for peptide loading has been addressed by comparative studies of HLA-B*44:02 and B*44:05 which differ only at position 116 (Asp for B*44:02 and Tyr for B*44:05) (88–90). These analyses suggested that HLA-B*44:05, which is tapasin-independent, preserves a peptide-free structure close to that of the peptide bound, even in the absence of tapasin.

A role for MHC-I conformational dynamics has been proposed to explain the differential disease susceptibility associated with two closely related HLA-B subtypes B*27:05 and B*27:09. Although the only amino acid sequence difference between the two subtypes is at position 116 in the floor of the peptide binding groove, which is Asp in B*27:05 and His in B*27:09, only B*27:05-expressing individuals are susceptible to ankylosing spondylitis (AS). Crystal structures of the two subtypes in complex with the same peptide are virtually identical. Using time-resolved fluorescence depolarization and MD simulations, Pohlmann et al. (91) showed that only peptide bound to the AS-associated subtype B*27:05 showed increased dynamics which is linked to the polymorphism at residue at 116. Thus, the increased dynamics is consistent with a molecule that has multiple conformational species that may aggregate either intra- or extracellularly contributing to various pathways to inflammatory disease.

Another point of difference between the B*27:05 and the B*27:09 subtypes is the dynamics at the β_2_m-heavy chain interface revealed by NMR. Using isotopically labeled human β_2_m, Beerbaum et al. (92, 93) compared the β_2_m-heavy chain interface in the two closely related HLA-B subtypes, complexed with four different peptides, and found significant chemical shift differences in a β_2_m loop that abuts the underside of the peptide binding groove and includes residues Asp53, Lys58, and Trp60. The most significant of these chemical shift differences is at Trp60 which shows subtype- and peptide-dependent structural variability. Conformational flexibility of β_2_m at the interface with heavy chain, revealed by NMR, may thus influence peptide binding affinities and consequently MHC-I stability at the surface with important functional consequences for T cell and NK cell recognition. In addition, molecules that facilitate MHC-I peptide exchange and loading, such as tapasin and TAPBPR, may employ recognition of this β_2_m-loop as a strategy to sense peptide occupancy, as discussed below.

Monoclonal antibodies that specifically recognize PR MHC-I molecules are valuable tools for identifying structural features that correlate with the conformation of the PR state. Among the best studied examples is the 64-3-7 antibody which binds to immature, PR H2-L^d^ but not to mature, PL H2-L^d^ (94). The minimal epitope of 64-3-7 is a sequence of seven amino acids in the H2-L^d^ α1 domain that adopts a 3_10_-helical conformation. Combining crystallographic, docking, and MD approaches, Mage et al. (95) showed that this 3_10_ helix moves in a hinge-like manner from an exposed and open position in the PR state to a closed position in PL molecules. The inward movement of the 3_10_ helix helps to form the A and B pockets that are crucial for stable peptide binding and subsequent release from tapasin in the PLC. It is noteworthy that the conformational dynamics of the 3_10_ helix occur at the opposite end of the groove from the site of tapasin binding—an illustration of the coordinated and dynamic changes that accompany peptide binding and chaperone release.

While computationally expensive, a wealth of information on the conformational flexibility of both peptide-bound and PR MHC molecules has been provided from all-atom MDs simulations in explicit solvent. In particular, in the absence of the peptide ligand, MHC-I molecules, such as HLA-A*02, HLA-B*44, HLA-B*27, H2-D^d^, H2-D^b^, and H2-K^b^, exhibit increased mobility in the F-pocket region of the MHC, adjacent to the α2-1 helix (11, 84, 85, 96–99). Peptide-dependent dynamic coupling between the heavy chain groove and the α3/light chain interface has also been observed (100–102). Likewise, MD has uncovered similar conformational flexibility in the opposite end of peptide-deficient class II MHC molecules (HLA-DR1 and HLA-DR3) at the α51–59 and β58–69 regions (83, 103, 104). Finally, a putative role for N-linked glycosylation in modulating the local flexibility of the MHC groove has also been explored (105). Together, these studies show that polymorphisms within the MHC groove may dictate both ligand binding and overall allotype stability through alteration in dynamics, either in localized regions or globally. Taken together, these data indicate that modulation of MHC dynamics plays a defining role in peptide exchange, stability at the cell surface and co-receptor engagement where sparsely populated transient states may be involved (106).

## Dynamics of Proteins of the Antigen Presentation Pathway

Newly synthesized MHC molecules are stabilized in a PR form in the PLC until loaded with high affinity peptide cargo. Following successful peptide loading, MHC-I molecules are released from the PLC and are transported through the Golgi to the cell surface. The PLC is a multimolecular, ER-membrane anchored assemblage consisting of the MHC-I/β_2_m complex itself, the lectin calreticulin, the transporter TAP1/TAP2, the chaperone and peptide editor tapasin, and the disulfide isomerase ERp57 (see Figure 1). The molecular organization of this complex has been deduced from biochemical experiments (43, 44, 46), and X-ray structures of the individual proteins (107–111). Recently the structure of the PLC was visualized by cryo-EM (49) revealing an arrangement of the component proteins that is consistent with previous biochemically derived structural models (27, 104, 112), which indicate the association of a central TAP heterodimer with two peptide editing modules, each consisting of calreticulin, ERp57, tapasin and MHC-I. In addition, the cryo-EM images revealed intermediate states that lacked calreticulin and/or MHC-I, affirming the transient and dynamic nature of the molecular interactions within the PLC. A key component of the PLC is tapasin whose importance in selective loading of MHC-I with high affinity peptides is illustrated by the greatly reduced cell surface levels of MHC-I in tapasin-deficient cell lines (113, 114) and mutant mice (77). Binding to tapasin stabilizes MHC-I molecules that are peptide-free or suboptimally loaded (115) until an appropriate high affinity peptide is bound leading to tapasin dissociation from the complex.

Detailed mechanistic understanding of tapasin function in peptide loading is lacking because the structure of a PR MHC-I in complex with tapasin has proved elusive. Nevertheless, the structure of a tapasin–ERP57 complex combined with extensive mutagenesis data has revealed structural details of tapasin function (111). Tapasin is an L-shaped protein consisting of a membrane proximal Ig-domain and an N-terminal domain that is a fusion of a β-barrel and an Ig-domain. Differences in the orientation of the tapasin N- and C-terminal domains in the three copies in the asymmetric unit suggests interdomain flexibility is a structural feature of tapasin. The MHC-I interaction sites on tapasin, inferred from extensive mutagenesis data, reveal an evolutionarily conserved, extensive binding interface encompassing residues on both of the tapasin Ig domains. Combining MDs simulations of peptide-free MHC-I (84) and mutagenesis data identifying tapasin binding sites on MHC-I (116, 117), a structural model of the tapasin/MHC-I was proposed in which the primary focus of tapasin is the short helical segment of the MHC-I, α2-1, which is conformationally mobile and sensitive to groove occupancy (111).

More recently, mechanistic insights into MHC-I peptide loading and glimpses of the conformational dynamics involved have been obtained by crystal structures of the tapasin-like molecule, TAPBPR, in complex with PR forms of MHC-I (10, 12). Like tapasin, TAPBPR is widely expressed, interferon-γ inducible (118), and catalyzes the loading of high affinity peptides (119, 120). However, unlike tapasin, TAPBPR is not associated with the PLC (118) and TAPBPR-deficient cell lines display normal levels of MHC-I (119). Also, unlike tapasin, TAPBPR is not found only in the ER but also in the cis-Golgi (118). Although the role of TAPBPR in antigen presentation and its functional relationship to tapasin remain enigmatic, TAPBPR may function downstream of the PLC in conjunction with UDP-glucose:glycoprotein glucosyltransferase (UGGT1) (121), to provide additional peptide quality control.

**Figure 2:**
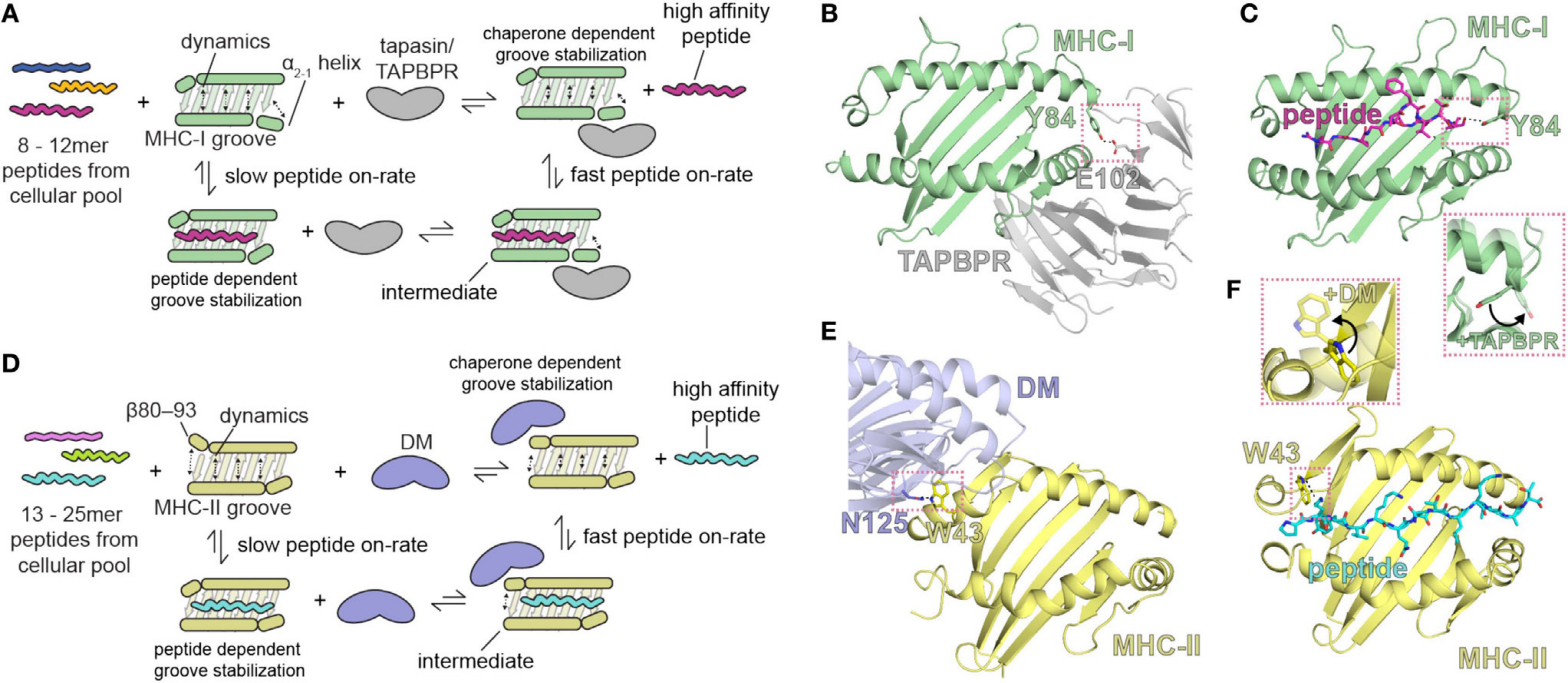
Summary of the role of major histocompatibility complex (MHC) groove structure and dynamics during peptide exchange and editing by the molecular chaperones tapasin or TAP-binding protein, related (TAPBPR). Schematic for class I **(A)** and class II **(D)** chaperone-assisted peptide exchange. Peptide exchange/editing occurs when peptides of 8–12 (class I) or 13–25 (class II) length are selected from the cellular pool in a manner highly dependent on the peptide sequence and the particular MHC allelomorph. This occurs either intrinsically (left) or is mediated by molecular chaperones (right) that increase the kinetic on-rate of peptides binding to the MHC groove through a stable intermediate complex. Conformational dynamics in localized regions of the MHC groove are stabilized by both the peptide and by molecular chaperones. The localized, dynamic regions including the α2-1 helix of MHC class I (MHC-I) and the β80-93 helix of MHC class II (MHC-II). **(B,E)** Structure of chaperone-bound MHC molecules with a focus on the MHC groove. Salmon dotted boxes highlight the conserved residues that are involved in chaperone-induced widening of the MHC groove (DM αN125 and MHC-II αW43; TAPBPR E102 and MHC-I Y84) relative to the peptide-bound state. **(C,F)** Structure of the peptide-bound (unchaperoned) MHC groove. Salmon dotted boxes highlight “flipping” of MHC-I Y84 and MHC-II W43 upon chaperone association, which in the absence of chaperones associate with the termini of the peptide. The chaperone-bound MHC states are shown as transparent in the inlays. PDB IDs are 5WER (H2-D^d^/TAPBPR), 3ECB (P18/H2-D^d^), 4FQX (HLA-DM/HLA-DR1), and 1DLH (Flu peptide/HLA-DR1).

The comparison of the three structures of H2-D^d^: (1) occupied by a truncated, suboptimal peptide (pdb: 5WES); (2) peptide-free, stabilized by TAPBPR (pdb: 5WER); and (3) complexed with a high-affinity peptide (pdb: 3ECB) illustrates the conformational rearrangements that accompany the transition of MHC-I from a partially PL complex to a peptide-receptive and then to a PL state (10). In the TAPBPR-stabilized PR form, the MHC-I groove is widened in the region of the F pocket due to an ~3 Å displacement of the α2-1 helical segment. In addition, β strands 5 and 8 that line the floor of the binding groove are displaced downward. The side chain of the conserved Tyr84 of MHC-I which in almost all pMHC-I structures coordinates both the C terminus of the peptide and Lys146 in the α2-1 helix is now flipped out of the groove to interact instead with Glu102 of TAPBPR (Figures 2B,C). Surprisingly, structural remodeling also occurs at the opposite end of the peptide binding groove as seen in the interaction between the side chains of Arg66 and Tyr159 which effectively close off this portion from peptide interaction. Extensive movements of the α3 domain and β_2_m subunit also illustrate the differences between the PR and PL states. The 58–60 loop of β_2_m which abuts the peptide binding platform from below and is conformationally dynamic and peptide-sensitive (92) forms key contacts to a hairpin loop of TAPBPR suggesting that peptide occupancy is sensed by TAPBPR through interaction with this β_2_m loop. As modeled in the TAPBPR/H2-D^b^ structure (12), peptide occupancy may also be sensed, and peptide loading facilitated, by a helix or loop of TAPBPR projecting into the groove near the F pocket. Finally, TAPBPR, like tapasin, has been suggested to stabilize the peptide-deficient MHC groove by dampening mobility of the α2-1 helix (122). Thus, as illustrated by both structures, coordinated and dynamic structural changes, stabilized transiently by TAPBPR interactions, occur during the critical step of MHC-I peptide loading.

A recent solution NMR study of the effects of the binding of TAPBPR to MHC-I (11) reveals stabilization of the dynamics of the empty MHC-I. On exposure to peptide, and with progressive peptide occupancy, the dynamics are further dampened, leading to an inverse relationship between MHC-I peptide occupancy and TAPBPR/MHC-I affinity. The NMR data reveal not only the interaction of conserved surfaces on the MHC-I heavy chain including the floor of the binding groove, the α2-1 helix, and the CD8 recognition loop of the α3 domain, but also effects on the α_1_ helix opposite the TAPBPR/tapasin binding site (and analogous to the HLA-DM binding site on MHC-II), all of which contribute to the widening of the binding groove in the chaperone-complexed but PR form of the MHC-I molecule. These results support a negative allostery release cycle as illustrated in Figure 2. In this mechanistic model, related in part to dynamics of the groove, the kinetic association rate of peptide binding to MHC-I is slow in the absence of a chaperone like tapasin or TAPBPR, and a peptide-receptive conformation is stabilized by the binding of the chaperone (Figure 2A). High affinity peptide binds rapidly to chaperone stabilized MHC-I, which ultimately releases the chaperone. A similar model is proposed for MHC-II binding to peptides, but in this case, the chaperone HLA-DM stabilizes the PR form of MHC-II by binding at the 3_10_ helix region (residues of the MHC-II β chain 80–93) (Figures 2A,E,F).

A crucial step in the peptide-loading process is the trimming of peptides by the ERAP1 aminopeptidase (ERAAP in the mouse), the importance of which is underlined by the antigen presentation defect of ERAAP-deficient mice (47). Recent studies, exploiting both crystallography and MD simulations indicate the critical role of dynamic changes for the aminopeptidase activity of ERAP1 (123, 124).

## Dynamic Aspects of T Cell Recognition of pMHC Complexes

Once pMHC complexes have arrived at the surface of the APC, they are available for recognition by TCR or NKR. Most of our understanding of the molecular details by which TCR on the T cell or NKR on NK cells engage pMHC on APCs derives from crystallographic studies of TCR/pMHC (125) or NK/pMHC complexes (6). Pioneering efforts to explore dynamic aspects of the TCR/pMHC interaction used NMR chemical shift analysis to map the footprint of a pMHC-specific TCR onto its cognate MHC (126). These studies employed a truncated MHC-I molecule to identify chemical shift perturbations in solution that resulted from binding to a single chain TCR ligand (also ~25 kDa). The binding footprint obtained in solution in this manner was the same as that determined crystallographically for the same complex. In a complementary set of experiments, using NMR to examine residues of the same 2C TCR and of a labeled peptide in the pMHC complex, Hawse et al. (60) explored the dynamic changes that accompany the interaction of the pMHC with the TCR. They showed that structural fluctuations of the pMHC ligand matched similar fluctuations of the TCR, suggesting that TCR use these dynamic changes in solution to scan through different pMHC and to match those that have similar flexible modes.

## TCR Changes That Accompany pMHC Interaction and Communicate Signal Transduction

**Figure 3:**
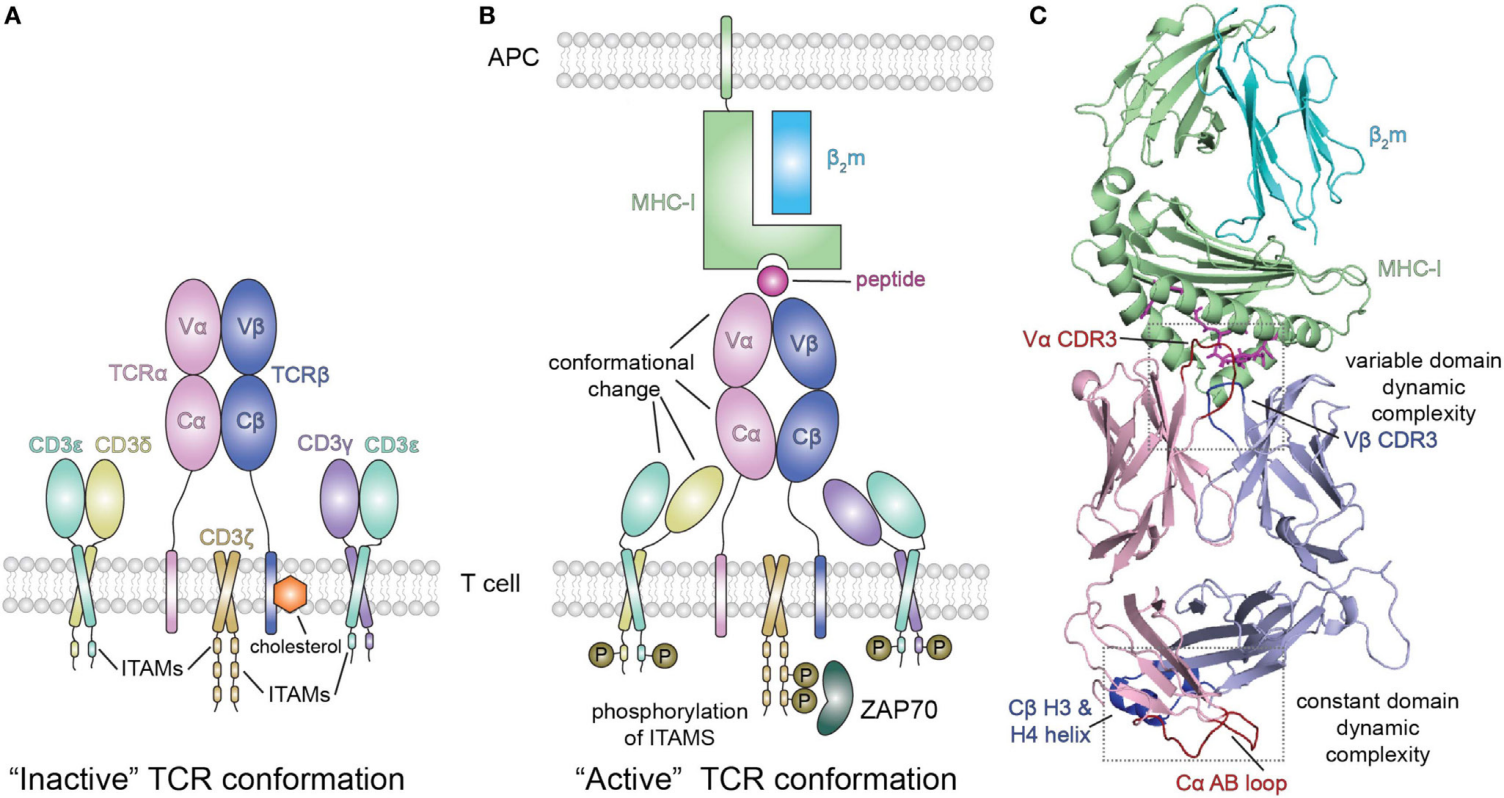
Peptide/MHC-I (pMHC-I) induced allosteric communication model for signaling of the TCR-CD3 complex. **(A)** In the absence of peptide–MHC-I ligand, cholesterol associates with the T cell receptor (TCR) β chain and maintains the TCR/CD3 complex in an “inactive” TCR conformation. The TCR αβ heterodimer is non-covalently associated with the CD3 γε and δε subunits as well as the ζζ homodimer. **(B)** Binding of pMHC-I molecules with the membrane distal variable domains (Vα, Vβ) of the αβ TCR receptor has been proposed to allosterically modulate the structure and dynamics of the membrane proximal constant domains (Cα, Cβ) which are sensed by the associated CD3 molecules resulting in an “active TCR.” This pMHC-I/TCR mediated conformational change in CD3 allows for phosphorylation (P) of downstream immunoreceptor tyrosine-based activation motifs (ITAMs) that recruit proteins involved in signaling, such as ZAP70. Abbreviation: APC, antigen-presenting cell. **(C)** Structural view of the extracellular domain of the bound pMHC-I/TAP-binding protein, related complex (PDB ID 5IVX). Gray dotted boxes highlight regions of dynamic complexity that are proposed to be involved in the allosteric communication model.

In addition to studies of the pMHCpMHC interaction and TCR/pMHC interaction noted above, several groups have addressed the mechanism by which pMHC engagement by a TCR may contribute to signal transduction (Figures 3A,B). The TCR, in addition to consisting of αβ chains that recognize the pMHC, contains the ζζ homodimer, and the CD3γε and CD3δε heterodimers, as part of an eight-chain complex embedded in the T cell membrane. Cytoplasmic immunoreceptor tyrosine-based activation motifs (ITAMs) extend from ζ, γ, δ, and ε and, when phosphorylated by the lymphocyte-specific protein tyrosine kinase (Lck), direct an activation cascade in the T cell. Aivazian and Stern (127) explored the lipid interaction of non-phosphorylated ζ chain and its mobilization from lipid vesicles when phosphorylated, suggesting that the availability of the ITAM for kinase activity was an early step in the extracellular binding of the eight chain TCR complex by a pMHC ligand. The structure of the cytoplasmic domain of the ζ chain has been explored in detergent micelles of LMPG and suggests that ITAM2 and ITAM3 interchange on the micro to millisecond timescale to regulate their accessibility for phosphorylation (128). Likewise, dynamic membrane associations that render the ITAM tyrosines inaccessible have been reported for the CD3ε cytoplasmic domain (129).

In efforts to explore the mechanism by which extracellular, cell-surface binding events convey conformational changes to cytoplasmic protein modules, three groups have explored changes in the CD3 (ζζ, γε, and δε) components of the TCR complex (13–15). He et al. (15) used an MHC-II-restricted αβ TCR isotopically labeled in the β chain to examine NMR chemical shift differences on exposure to γε and/or δε heterodimers. They observed small differences in a set of 9–11 solvent accessible Cβ residues consistent with a docking site requiring both γε and δε.

Using a different MHC-II-restricted TCR, others (14) labeled either the α or β chain and identified NMR spectroscopic changes in the constant regions consistent with δε docking on the Cα domain and γε on Cβ. These results were further supported by functional studies of mutagenized TCR in transfected T cells.

Extending this approach, Natarajan et al. (13) used a high affinity MHC-I restricted TCR to examine changes in the β chain TCR spectrum on pMHC binding. Remarkably, in addition to the dynamic changes of the interface residues of the TCR [the complementarity determining residues (CDRs)], these authors observed significant chemical shift changes in regions of the TCR remote from the pMHC interface, in particular near the Cβ H3 and H4 helices (Figure 3C). Confirmatory evidence was provided by site-directed mutagenesis and functional assays, consistent with an allosteric effect in the constant region resulting from pMHC-I engagement. The authors suggest that the allosteric transmission of conformational changes from the TCR CDRs in the variable domain to the Cβ distal sites occurs *via* the modulation of the variable/constant domain interface through the structural or dynamic rearrangement of the Vβ/Cβ linker regions.

## Conclusion

Biochemical evidence has long suggested that dynamic aspects of MHC molecules, the chaperones of the PLC, and the interactions with TCR might contribute to aspects of the functional molecular recognition steps throughout the entire MHC antigen presentation pathway. Only in the last few years have the combination of high resolution structural studies, computational MD, and multidimensional NMR been applied together to generate a mechanistic view of how conformational plasticity and MDs regulate multiple steps along the antigen processing and presentation pathway. It is now clear that molecular flexibility in peptide loading onto MHC, MHC/chaperone interaction, and pMHC interaction with TCR form a set of dynamic events contributing to their biological and potentially pathogenic role. A classical view of protein structure/function relationships ascribes function to the most stable (lowest energy) conformation. This understanding is being challenged by our appreciation that molecules that exhibit exceptional conformational diversity, known as intrinsically disordered proteins (IDPs) can represent a mixture of structured and unstructured regions or may even be entirely unstructured (130, 131). As a result, IDPs function by virtue of molecular associations that disregard traditional lock-and-key requirements and show flexibility in ligand binding. Studies of antigenic peptide dynamics, MHC-I and -II conformation changes, chaperone interactions, and pMHC-dependent TCR allostery now begin to reveal how dynamic or disordered regions of proteins contribute to their biological function. We expect that further studies of the molecular and cellular details of antigen processing, presentation and T cell signaling will shed light not only on this central aspect of the immune response, but also contribute to a more comprehensive understanding of how protein sequence, structure, and dynamics shape the biological function of macromolecules in general.

## A Final Word

This review summarizes some of the enormous progress that the immunological community as a whole has made in addressing fundamental mechanisms of molecular recognition that initiate and propagate immune responses. Nevertheless, there remain complexities yet to be revealed as our understanding evolves from the specific to the general. Bill Paul had the unique ability to identify central problems whose solutions then would stimulate whole new areas of investigation. We trust that this review reflects in small part his continuing influence in encouraging us to study important questions and to seek definitive answers.

“I believe that a leaf of grass is no less than the journey-work of the stars.”*Leaves of Grass*, Walt Whitman

## Author Contributions

KN, JJ, NAM, MGM, LFB, ACM, NGS, AB, and DHM conceived and wrote various parts of the review according to their expertise. KN, ACM, NGS, AB, and DHM edited and assembled the various contributions to the final text.

## Conflict of Interest Statement

The authors declare that the research was conducted in the absence of any commercial or financial relationships that could be construed as a potential conflict of interest. The reviewer YS and handling Editor declared their shared affiliation.
